# Feasibility of enhanced recovery after surgery in gastric surgery: a retrospective study

**DOI:** 10.1186/1471-2482-14-41

**Published:** 2014-07-08

**Authors:** Takanobu Yamada, Tsutomu Hayashi, Toru Aoyama, Junya Shirai, Hirohito Fujikawa, Haruhiko Cho, Takaki Yoshikawa, Yasushi Rino, Munetaka Masuda, Hideki Taniguchi, Ryoji Fukushima, Akira Tsuburaya

**Affiliations:** 1 Department of Gastrointestinal Surgery, Kanagawa Cancer Center, 1-1-2 Nakao, Asahi, 241-0815 Yokohama, Kanagawa, Japan; 2 Department of Surgery, Yokohama City University, Yokohama, Japan; 3 Department of Anesthesiology, Kanagawa Cancer Center, 1-1-2 Nakao, Asahi, 241-0815 Yokohama, Kanagawa, Japan; 4 Department of Surgery, Teikyo University School of Medicine, 2-11-1 Kaga, 173-8605 Itabashi, Tokyo, Japan

**Keywords:** Enhanced recovery after surgery, Gastric cancer, Gastrectomy, Feasibility, Postoperative complications

## Abstract

**Background:**

Enhanced recovery after surgery (ERAS) programs have been reported to be feasible and useful for maintaining physiological function and facilitating recovery after colorectal surgery. The feasibility of such programs in gastric surgery remains unclear. This study assessed whether an ERAS program is feasible in patients who undergo gastric surgery.

**Methods:**

The subjects were patients who underwent gastric surgery between June 2009 and February 2011 at the Department of Gastrointestinal Surgery, Kanagawa Cancer Center. They received perioperative care according to an ERAS program. All data were retrieved retrospectively. The primary end point was the incidence of postoperative complications. The secondary end point was postoperative outcomes.

**Results:**

A total of 203 patients were studied. According to the Clavien-Dindo classification, the incidence of ≥ grade 2 postoperative complications was 10.8% and that of ≥ grade 3 complications was 3.9%. Nearly all patients did not require delay of meal step-up (95.1%). Only 6 patients (3.0%) underwent reoperation. The median postoperative hospital stay was 9 days. Only 4 patients (2.0%) required readmission. There was no mortality.

**Conclusions:**

Our results suggest that our ERAS program is feasible in patients who undergo gastric surgery.

## Background

Gastric cancer is the second leading cause of cancer-related death worldwide [1]. Complete surgical resection plays the most important role in the cure of gastric cancer; however, surgery for gastric cancer remains a high-risk procedure with clinically significant postoperative stress, complications, and sequelae. Morbidity and mortality from radical gastrectomy range from 9.1-46.0% and 0-13%, respectively [2-7].

Enhanced recovery after surgery (ERAS) programs have been proposed to maintain physiological function and facilitate postoperative recovery [8]. In the following studies, ERAS was considered to reduce rates of morbidity, shorten length of hospital stay [9-11]. ERAS programs have many elements, including preoperative education, preoperative carbohydrate loading, omission of bowel preparation, epidural analgesia without opioids, early postoperative enteral feeding, early mobilization of patients, and thrombotic prophylaxis. In colorectal surgery, several studies have reported that ERAS programs are feasible and useful [9-11].

We previously demonstrated that an ERAS protocol is useful in patients who undergo elective radical gastrectomy [12]. Other studies have reported that ERAS programs or fast-track surgery in gastric surgery can accelerate postoperative rehabilitation [13-18]. However, the number of patients assessed in these studies was small. In addition, some studies found that the incidence of some postoperative complications, especially nausea, vomiting, and postoperative ileus, tended to be higher in the ERAS group than in the conventional group, suggesting that ERAS programs might increase the risk of some complications after gastric surgery. Because of these controversial results, it is necessary to confirm the feasibility of ERAS programs in gastric surgery.

The present study evaluated whether an ERAS program was feasible in more than 200 patients who underwent gastric surgery. Emphasis was placed on postoperative gastrointestinal complications.

## Methods

### Patients

Between June 2009 and February 2011, a total of 256 patients with gastric cancer underwent surgery at the Department of Gastrointestinal Surgery, Kanagawa Cancer Center. The inclusion criteria for this study were (1) a histologically confirmed diagnosis of adenocarcinoma of stomach and (2) the receipt of elective distal gastrectomy or total gastrectomy with lymphadenectomy. We excluded patients who preoperatively received any chemotherapy or radiotherapy. We also excluded patients who underwent proximal gastrectomy or laparoscopy-assisted total gastrectomy because these procedures are not standard in Japan and are mainly used in patients enrolled in clinical trials. These surgeries themselves have not been confirmed safe and feasible yet.

All patients received perioperative care according to our ERAS program. Operations were performed by the same team of surgeons (three specialists and four trainees). In principle, patients with a preoperative diagnosis of stage I disease received laparoscopic surgery with D1+ dissection, and the others received open surgery with D2 dissection [19].

### ERAS program

In their Cochrane review, Spanjersberg *et al*. regard ERAS protocols as programs that include 7 or more of 17 ERAS items [10]. Our ERAS program included 13 items.

#### ***Preoperative***

Preoperative counseling was held in the outpatient clinic before hospitalization and in the ward after admission. Patients could eat a normal diet until dinner of the day before surgery. Magnesium oxide and a New Lecicarbon^®^ suppository (Zeria Pharmaceutical Co., Ltd., Tokyo, Japan) were administered on the day before surgery (Table 1).

**Table 1:** Time table of the modified ERAS program

Operative day	-1	0	+1	+2	+3	+4	+5	+6	+7
Preoperative counseling	Preoperative counseling was held in the outpatient clinic before hospitalization and in the ward after admission.								
Pre-medication	Patients do not receive any sedation.								
Oral intake	Normal diet until midnight	Oral hydration solution (OS-1^®^) 3 h before surgery		Drink a water and oral nutrition supplement (Endure Liquid^®^)	Liquid diet (3 steps up to a soft diet every 2 days)				
Bowel preparation	1 g magnesium oxide and a New Lecicarbon^®^ suppository								
Anesthesia and Analgesics		Combination of epidural analgesia (TH7-11) and general anesthesia during surgery							
		Continuous thoracic epidural infusion of analgesics after surgery	→	Removing epidural catheter					
		Nonsteroidal anti-inflammatory drug intravenously after surgery twice daily	→	Acetoaminophen three times a daily orally	→	→	→		
Drain and NGT		No drain in distal gastrectomy, one or two drains in total gastrectomy		Removing drain(s)					
		NGT was removed immediately after surgery							
Urinary catheter				Removing					
ADL			Encourage to sit out of bed for more than 6 h	Encourage to walk the length of the ward	→	→	→	→	→
Antithromboprophylaxis			None	Subcutaneous injection of antithrombotic agent (enoxaparin sodium or fondaparinux)	→	→	None	→	→
X-ray and blood exam.		○	○						○ (Check discharge creiteria)

NGT: Nasogastric tube, ADL: Activity of daily life.

#### ***Perioperative***

Patients, excluding those who had gastric obstruction with decreased output, could drink two 500-ml plastic bottles of OS-1^®^ (2.5% carbohydrate, Otsuka Pharmaceutical, Tokushima, Japan) 3 h before surgery. Premedication was not administered. Anesthesia consisted of a combination of epidural analgesia (Th 7-11) and general anesthesia. In principle, no drain was used in distal gastrectomy, and one or two drains were used in total gastrectomy. The nasogastric tube was removed immediately after surgery (Table 1).

#### ***Postoperative***

Day of surgery: A continuous thoracic epidural infusion of analgesics was given for 2 days after surgery. To prevent postoperative pain, a nonsteroidal anti-inflammatory drug (50 mg flurbiprofen axetil) was administered intravenously twice daily after surgery until the resumption of oral intake. Postoperative day (POD) 1: Patients were encouraged to sit out of bed for more than 6 h. POD 2: Oral intake was started with water and a can of oral nutrition supplement (250 ml Ensure Liquid ^®^, Abbott Japan Co., Ltd., Tokyo, Japan). After the resumption of oral intake, 300 mg of acetaminophen was administered orally three times daily. The patients were encouraged to walk the length of the ward. An antithrombotic agent (enoxaparin sodium 2 000 IU twice daily or fondaparinux 2.5 mg daily) was injected for 2 days 6 h after removal of the epidural catheter. POD 3: The patients started to eat soft food and were stepped up to regular food every 2 days (3 steps). The criteria for discharge were as follows: adequate pain relief, soft diet intake, return to preoperative mobility level, and normal laboratory data on POD 7 (Table 1).

The ERAS program evaluated in the present study was developed by a team of surgeons and anesthesiologists working in close cooperation with a data safety monitoring committee (DSMC). The feasibility and safety audit was completed by the DSMC in September 2009, when 50 patients had been treated according to the ERAS program. Our ERAS program in practice was approved both by the institutional clinical pathway committee and the DSMC. This study, a retrospective analysis, have been performed upon the approval of the institutional review board of Kanagawa Cancer Center. Informed consent for the ERAS program and using the clinical date without identifying personal information were taken before surgery.

### Data collection (end points)

All data were retrieved retrospectively from the patients’ database and clinical records. The primary end point was the incidence of postoperative complications. Complications were defined as ≥ grade 2 complications according to Clavien-Dindo classification within 30 days after surgery [20]. Secondary end points were postoperative outcomes such as onset of walking, onset of oral intake, onset of flatus, onset of defecation, delay of meal step-up, reoperation, postoperative hospital stay, readmission, and mortality. We delayed meal step-up if the patient ate 40 percent or less of meals for 2 days.

Pathological findings were categorized according to the 7^th^ edition of UICC-TNM [21]. Continuous data are expressed as medians (range).

## Results

A total of 203 patients were studied.

### Patients’ characteristics

There were 133 men and 70 women. Ten patients diagnosed non-adenocarcinoma or having other cancer simultaneously, sixteen patients received preoperative chemotherapy, Twenty two patients undergoing laparoscopy-assisted total gastrectomy, four patients undergoing remnant total gastrectomy, and one patient undergoing proximal gastrectomy were excluded. The median age was 67 (32-84) years. Performance status was good in most patients. Mean body mass index (BMI) was 22.2 (16.2-31.4) (Table 2).

**Table 2:** Clinicopathological features

		**Patients(N = 203)**	
Age (years old)^*^		67(32-84)	
Gender	Male	133	(65.5%)
	Female	70	(34.5%)
BMI (kg/m2)^*^		22.2(16.2-31.4)	
ECOG-PS	0	177	(87.2%)
	1	26	(12.8%)
	2	0	(0.0%)
ASA-PS	I	99	(48.8%)
	II	103	(50.7%)
	III	1	(0.5%)
Procedure	Open distal gastrectomy	67	(33.0%)
	Laparoscopy-assisted distal gastrectomy	76	(37.4%)
	Open total gastrectomy	60	(29.6%)
Lymph node dissection	D1,D1+	120	(59.1%)
	D2	83	(40.9%)
Reconstruction	Billroth I	116	(57.1%)
	Billroth II	4	(2.0%)
	Roux-en Y	83	(40.9%)
Combined organ resection	yes	34	(16.7%)
	no	169	(83.3%)
Operation time (min.)^*^		179(80-577)	
Bleeding (ml)^*^		110(0-1620)	
T categories	T1	121	(59.6%)
	T2	27	(13.3%)
	T3	19	(9.4%)
	T4	36	(17.7%)
N categories	N0	131	(64.5%)
	N1	22	(10.8%)
	N2	19	(9.4%)
	N3	31	(15.3%)
Stage (UICC TNM 7th)	I	128	(63.1%)
	II	35	(17.2%)
	III	31	(15.3%)
	IV	9	(4.4%)

BMI; Body mass index, ECOG-PS; Eastern cooperative oncology group performance status, ASA-PS; American society of anesthesiologists physical status, *median(range).

### Surgical procedures

Laparoscopic surgery was performed in 76 patients (37.4%), and total gastrectomy in 60 patients (29.6%). 83 patients (40.9%) underwent D2 lymph node dissection. Combined organ resection was performed in 34 patients, among whom 29 (85.3%) additionally underwent splenectomy (including pancreatico-splenectomy in 1 patient). Billroth I reconstruction was performed in 116 (57.1%) patients, and Roux-en-Y reconstruction was done in 83 (40.9%). The median operation time was 179 (80-577) minutes. The estimated median blood loss was 110 (0-1620) ml (Table 2).

### Pathological characteristics

Early gastric cancer (T1) was confirmed in 121 patients (59.6%). 131 patients (64.5%) had no evidence of lymph node metastasis. Final disease stage was stage I in 128 patients (63.1%), stage II in 35 (17.2%), stage III in 31 (15.3%), and stage IV in 9 (4.4%) (Table 2).

### Complications and postoperative course

Grade 2 or higher complications developed in 23 (11.3%) patients, and grade 3 or higher complications developed in 8 (3.9%). Anastomotic leakage occurred in 3 patients (1.5%), ileus in 2 patients (1.0%), and anastomotic stenosis in 2 patients (1.0%). The incidence of ≥ grade 2 complications in patients who underwent open distal gastrectmy, laparoscopy-assisted distal gastrectomy, or open total gastrectomy were 1.9%, 3.4%, or 5.4% respectively (p = 0.069). The incidence of ≥ grade 2 complications in patients who underwent D1 or D1+ dissection was 5.8%, as compared with 18.1% in patients who underwent D2 dissection (p = 0.010). The median onsets of oral intake, flatus, and defecation were POD 2 (1-5), 2 (0-5), and 4 (2-9), respectively. The step-up schedule for meals was delayed in 10 (4.9%) patients. Five of these patients (2.4%) had inadequate oral caloric or fluid intake because of nausea or vomiting. Reoperation was performed in 6 (3.0%) patients. The reasons for reoperation were leakage of the duodenal stump in 2 patients, leakage of the gastroduodenal anastomosis in 1, bowel obstruction in 1, anastomotic stenosis in 1, and pyothorax in 1. The median postoperative hospital stay was 9 (7-53) days. 4 (2.0%) patients were readmitted within 30 days after surgery. The reasons for readmission were ascites, fever, poor oral intake, and surgical site infection in 1 patient each. There was no mortality. No patient had postoperative pneumonia or required replacement of a nasogastric tube, with exception of 3 patients who had postoperative ileus (Tables 3 and 4).

**Table 3:** Complications

	**Clavien-Dindo classification**	
	**≥grade 2 n (%)**	**≥grade 3 n (%)**
Pancreas fistula	7(3.4%)	2(1.0%)
Anastomotic leakage	3(1.5%)	3(1.5%)
Ileus	2(1.0%)	0(0%)
Anastomotic stenosis	2(1.0%)	1(0.5%)
Surgical site infection	2(1.0%)	0(0%)
Obstruction	1(0.5%)	1(0.5%)
Pyothorax	1(0.5%)	1(0.5%)
Bleeding	1(0.5%)	0(0%)
Pleural effusion	1(0.5%)	0(0%)
Cholangitis	1(0.5%)	0(0%)
Ascites	1(0.5%)	0(0%)
Toxicoderma	1(0.5%)	0(0%)
Total	23(11.3%)	8(3.9%)

**Table 4:** Postoperative outcomes

Onset of walk (POD^*^)	2(1-4)
Onset of oral intake (POD^*^)	2(1-5)
Onset of flatus (POD^*^)	2(0-5)
Onset of defecation (POD^*^)	4(2-9)
Delay of meal step up (n(%))	10(4.9%)
Complication CD ≥ grade2 (n(%))	23(11.3%)
Reoperation (n(%))	6(3.0%)
Postoperative hospital stay(days)^*^	9(7-53)
Readmission (n(%))	4(2.0%)
Mortality (n(%))	0(0%)

POD; Post operative day, CD; Clavien-Dindo classification, *median (range).

## Discussion

This is the first large study to evaluate the feasibility of a comprehensive ERAS program in gastric surgery. In our study, the overall incidence of morbidity (i.e., ≥grade 2 complications) was 10.8%, and the incidence of clinically significant events (≥grade 3 complications) was only 3.9%. These results obtained with our ERAS program are good as compared with complication rates of conventional perioperative care group in our previous report (12.0%) as well as other reported complication rates without ERAS program (10.5-46.0%) [2-7,12]. Although T1 tumors and D1 dissection were dominant in our study, the morbidity rate in the D2 group (18.1%) was comparable to that in a large randomized controlled trial performed in Japan (JOCG9501 study, 20.9%) [5].

Among the many elements of ERAS programs, one of the utmost concerns for surgeons is that early postoperative feeding can induce postoperative ileus or anastomosis leakage. Because of these concerns, oral intake of food was previously not allowed for several days after gastrectomy in Japan [22]. In some European countries, food is also withheld for several postoperative days [23], but this practice is not supported by adequate evidence. In fact, in our study the incidences of postoperative ileus and anastomotic leakage were very low (1.0% and 1.5%, respectively), as compared with previous studies (0-12.5% and 0-4.2%, respectively) [2-7]. The incidences of those in conventional prieoperative care of our hospital (0% and 2%, respectively) were similar with these results [12]. Furthermore, meal step-up did not have to be delayed in nearly all patients (95.1%), and this is also similar with previous reported our internal control (96.0%) [12]. Several studies have also demonstrated that early oral feeding is feasible and beneficial in gastric surgery [13-18,24,25], but this point remains controversial. Heslin *et al*. reported that early enteral feeding was not beneficial after surgery for upper gastrointestinal malignancies [26]. On the other hand, Lewis *et al*. found in their meta-analysis that keeping patients ‘nil by mouth’ is without benefit; in contrast, early enteral feeding was suggested to reduce mortality [27]. There were six complications that need reoperation in current study. Among those, possibility of relation between the leakage of the gastroduodenal anastomosis and ERAS program could not be denied. But others seem to be unlikely. Our results and the findings of previous studies suggest that early oral or enteral feeding is at least feasible and does not increase the risk of postoperative ileus or anastomotic leakage.

Our study had several limitations. 1) It was conducted retrospectively in a single hospital, and the analyses and endpoints were not preplanned. 2) Most patients had good performance status. Patients with poor performance status (e.g. Eastern cooperative oncology group performance status ≥3, severe dementia, and swallowing difficulty) could not be treated in our hospital, because we specialize in cancer treatment. These could be selection bias. 3) More than half of the patients had T1 disease and underwent limited lymph node dissection (D1+), whichmight account for the good results of our study. In particular, D2 or more radical lymph node dissection has been repeatedly reported to increase the risk of surgery-related complications [2-5]. Consistent with the previous findings, the incidence of complications was higher after D2 dissection than after D1 dissection in our study. Finally, 4) our ERAS program did not include fluid management, which is one of the key elements of ERAS programs. There was not robust evidence of perioperative fluid management in gastric surgery at the time we introduced ERAS.

## Conclusions

In conclusion, our results suggest that our ERAS program is feasible in at least early-staged and relatively young patients undergoing elective gastric surgery. Further studies, for which population ERAS program can be applied and whether ERAS programs have potential benefits, are necessary.

## Abbreviations

ERAS: Enhanced recovery after surgery; POD: Postoperative day; DSMC: Data safety monitoring committee; BMI: Body mass index.

## Competing interests

The authors declare that they have no competing interests.

## Authors’ contributions

TY, TH, TA, JS, HF, HC, TY, HT, RF, and AT conceived and coordinated the study, collected patients’ data and participated in the statistical analysis. RY, MM, and AT participated in preparing and drafting the manuscript. All authors read and approved the final manuscript.

## Pre-publication history

The pre-publication history for this paper can be accessed here:

http://www.biomedcentral.com/1471-2482/14/41/prepub
